# Prescribed and Penalized: The Detrimental Impact of Mandated Reporting for Prenatal Utilization of Medication for Opioid Use Disorder

**DOI:** 10.1007/s10995-023-03672-x

**Published:** 2023-05-31

**Authors:** Erin C. Work, Serra Muftu, Kathryn Dee L. MacMillan, Jessica R. Gray, Nicole Bell, Mishka Terplan, Hendree E. Jones, Julia Reddy, Timothy E. Wilens, Shelly F. Greenfield, Judith Bernstein, Davida M. Schiff

**Affiliations:** 1 Division of General Academic Pediatrics, MassGeneral for Children, 125 Nashua St Suite 860, Boston, MA 02114 USA; 2 Department of Community Health Sciences, UCLA Fielding School of Public Health, 650 Charles E. Young Dr. S., Los Angeles, CA 90095 USA; 3 Department of Social Welfare, UCLA Luskin School of Public Affairs, 337 Charles E. Young Dr. E., Los Angeles, CA 90095 USA; 4 Division of Newborn Medicine, Brigham and Women’s Hospital, Boston, MA 02114 USA; 5 Division of Pediatric Hospital Medicine, MassGeneral Hospital for Children, 55 Fruit St, Boston, MA 02114 USA; 6 Division of General Internal Medicine, Massachusetts General Hospital, Boston, MA 02114 USA; 7 Living in Freedom Together-LIFT Inc, Worcester, MA USA; 8 Friends Research Institute, 1040 Park Ave, Suite 103, Baltimore, MD 21202 USA; 9 UNC Horizons and Department of Obstetrics and Gynecology, University of North Carolina Chapel Hill, 410 North Greensboro St., Carrboro, NC USA; 10 Gillings School of Public Health, University of North Carolina Chapel Hill, Chapel Hill, NC USA; 11 Division of Child and Adolescent Psychiatry, Massachusetts General Hospital, 55 Fruit St, Boston, MA 02114 USA; 12 Division of Women’s Mental Health and Division of Alcohol, Drugs, and Addiction, McLean Hospital, 115 Mill St, Belmont, MA 02478 USA; 13 Harvard Medical School, 25 Shattuck Street, Boston, MA 02115 USA; 14 Department of Community Health Sciences, Boston University School of Public Health, Boston, MA USA; 15 Division of Pediatrics, MassGeneral for Children, Boston, USA

**Keywords:** Perinatal opioid use disorder, Medications to treat opioid use disorder, Mandated reporting, Child protection systems, Substance-exposed newborns

## Abstract

**Objectives:**

Some states, including Massachusetts, require automatic filing of child abuse and neglect for substance-exposed newborns, including infants exposed in-utero to clinician-prescribed medications to treat opioid use disorder (MOUD). The aim of this article is to explore effects of these mandated reporting policies on pregnant and postpartum people receiving MOUD.

**Methods:**

We used modified grounded research theory, literature findings, and constant comparative methods to extract, analyze and contextualize perinatal experiences with child protection systems (CPS) and explore the impact of the Massachusetts mandated reporting policy on healthcare experiences and OUD treatment decisions. We drew from 26 semi-structured interviews originally conducted within a parent study of perinatal MOUD use in pregnancy and the postpartum period.

**Results:**

Three themes unique to CPS reporting policies and involvement emerged. First, mothers who received MOUD during pregnancy identified mandated reporting for prenatally prescribed medication utilization as unjust and stigmatizing. Second, the stress caused by an impending CPS filing at delivery and the realities of CPS surveillance and involvement after filing were both perceived as harmful to family health and wellbeing. Finally, pregnant and postpartum individuals with OUD felt pressure to make medical decisions in a complex environment in which medical recommendations and the requirements of CPS agencies often compete.

**Conclusions for Practice:**

Uncoupling of OUD treatment decisions in the perinatal period from mandated CPS reporting at time of delivery is essential. The primary focus for families affected by OUD must shift from surveillance and stigma to evidence-based treatment and access to supportive services and resources.

**Supplementary Information:**

The online version contains supplementary material available at 10.1007/s10995-023-03672-x.

## Significance

*What is already known on this subject?* Child protection systems (CPS) reporting is associated with barriers to prenatal care and family resources and services. Some state policies in the United States mandate reporting to CPS for prenatal substance exposure, including prescribed medications for opioid use disorder.

*What this study adds?* This study centers the experiences of pregnant and postpartum people with opioid use disorder with mandated reporting policies for prenatal substance exposure, describes the harms to families associated with these policies, and makes recommendations for policy change. Findings emphasize the need to uncouple medical decisions from CPS reporting and involvement.

**Supplementary Information:**

The online version contains supplementary material available at 10.1007/s10995-023-03672-x.

## Introduction

Medications for opioid use disorder (MOUD), including methadone and buprenorphine, are recommended for treatment of opioid use disorder (OUD) in pregnancy (“Committee Opinion No. 711: Opioid Use and Opioid Use Disorder in Pregnancy,” 2017). However, pregnant patients with OUD are often hesitant to start or continue medications due to stigma, concern around neonatal opioid withdrawal syndrome (NOWS), and fear health care workers will report to child protection systems (CPS) after delivery (Guille et al., 2019; Ostrach & Leiner, 2019; Schiff et al., 2022). In the United States, federal legislation mandates that states track data on all substance-exposed newborns, including exposure to legal substances, and ensure that a “Plan of Safe Care” is created for each family (“CAPTA Reauthorization Act of 2010,” 2010; “Comprehensive Addiction and Recovery Act of 2016,” 2016). In response, the Massachusetts CPS agency, the Department of Children and Families (DCF), provided guidance that prenatal substance exposure—including prescribed MOUD, according to DCF—is an indication to file a report for alleged child abuse/neglect (Massachusetts Department of Children & Families, 2016).

Previous studies have explored several negative effects of CPS—referred to by critics as family regulation (Roberts, 2022)—surveillance and involvement on families. Apprehension about CPS involvement can deter individuals from engaging with resources that might link them to CPS (Fong, 2020). Mothers with a history of custody loss were more likely than other mothers to receive inadequate prenatal care in subsequent pregnancies (Wall-Wieler et al., 2019). Among pregnant and parenting individuals who use drugs or have a substance use disorder (SUD), fear of CPS involvement and custody loss contributes to avoidance of prenatal services (Roberts & Pies, 2011; Stone, 2015). When reporting leads to CPS involvement and child removal, custody loss has been associated with increased risk of recurrence of use and opioid-related overdose among mothers (Cleveland et al., 2020). Further, a meta-synthesis of studies investigating mandated reporters’ experiences found that harm to therapeutic relationships following child removal was common (McTavish et al., 2017). Despite these findings, there is little published on the effects of mandated reporting policies for prenatal substance exposure, including MOUD, focusing on the perspective of families.

Impacted families, researchers, and policymakers have identified the need for strategies to ensure pregnant people with SUD obtain prenatal care and SUD treatment without fear of child removal (McTavish 2017; Office of National Drug Control Policy, 2021; Rise Participatory Action Research Team, 2021). Massachusetts is a suitable study location due to comparitively high rates of prenatal MOUD utilization and its state mandated reporting policy (Massachusetts Department of Children & Families, 2016; Peeler et al., 2020). The objective of this study is to describe the perspectives of pregnant and postpartum people with OUD on state-mandated reporting for in-utero MOUD exposure.

## Materials and Methods

### Study Design

This study is a secondary analysis of a qualitative examination of experiences with MOUD engagement and adherence in the perinatal period (Schiff et al., 2022). The interview guide explored (1) substance use treatment, pregnancy, and delivery experiences; (2) personal and community attitudes toward MOUD; and (3) barriers and facilitators to treatment engagement and recovery. The guide (Supplemental document 1) was updated iteratively to include questions on emerging themes. The study was approved by the Partners Healthcare Institutional Review Board.

### Recruitment and Data Collection

Individuals with OUD and who had delivered a baby in the last three years were eligible for participation in semi-structured interviews between 2019 and 2020. Participants were primarily recruited from a multidisciplinary clinic specializing in care for families impacted by SUD in Boston, Massachusetts with additional recruitment through word of mouth. Two authors (DMS, JRG) were involved in the clinical care of many participants but did not participate in recruitment. Interviews lasted 30–90 min in-person or via phone or video before and during the COVID-19 pandemic. Participants received a $40 gift card for participation. Interviews were audio recorded and professionally transcribed. Full eligibility and recruitment methods for the parent study have been reported previously (Schiff et al., 2022).

### Data Analysis

A preliminary codebook was created using an inductive coding process with the first nine de-identified transcripts by four researchers (DMS, JAB, SBP, ECW) and then updated based on new findings. Interviews were conducted until thematic saturation was reached for the parent study’s aims. We utilized constant comparative methods in data collection and analysis to update the interview guide, code interviews, and develop themes concurrently (Taylor & Bogdan, 1998). The codebook was finalized by categorizing topics thematically and iteratively testing preliminary codes on transcripts. Two researchers independently coded the interviews in NVivo (ECW, SM). Discrepancies were reviewed until consensus was reached with a final kappa coefficient of 0.88 for all interviews. We reviewed data coded for mandated reporting and CPS involvement including “DCF involvement,” “disruption of normal parenting experience,” “loss of custody,” “mandated reporting,” “negative experiences with DCF,” “positive experiences with DCF,” “DCF perception of MOUD,” and “variability of DCF caseworkers” were separated for this secondary analysis. From these codes we generated three broad themes encapsulating participant experiences and selected illustrative quotes to demonstrate these themes. We used the Reproductive Justice framework to contextualize our findings, which includes a focus on the right to have and parent children and on the social, political, and economic inequities that affect access to reproductive decisions (Ross & Solinger, 2017). Our analysis addressed the impact of mandated reporting on participants’ experiences and identities as mothers and on their ability to autonomously engage with the healthcare and SUD treatment systems.

## Results

Twenty-six interviews were completed out of 31 potential participants approached; 5 declined or were lost to follow-up. Twenty-four participants were receiving MOUD at time of delivery (66.7% buprenorphine, 33.3% methadone). One interviewee was using non-prescribed substances at delivery and initiated methadone postpartum. Another participant discontinued buprenorphine during pregnancy to avoid CPS reporting and potential NOWS. She reinitiated buprenorphine two months postpartum and was the only participant without a CPS report filed directly following delivery. Of the 25 participants who had a CPS report filed at delivery, 24 (96%) were screened in for a full investigation. Twelve participants (46.2%) experienced child removal prior to the study interview. Six of the 12 participants who experienced child removal (50%) were later reunified with their child. Participant characteristics are shown in Table 1.

**Table 1:** Participant demographics

Characteristic	n (%) or mean (SD)
Age, mean (SD)	33 years (4.6)
Months from delivery when interviewed, mean (range)	10.1 months (3–33.1)
Race	
American Indian/Alaska Native	1 (3.8%)
Black or African American	3 (11.5%)
Mixed Race	3 (11.5%)
White	19 (73.1%)
Ethnicity	
Hispanic or Latina	5 (19.2%)
Non-Hispanic or Latina	21 (80.8%)
Sexual orientation	
Heterosexual	24 (92.3%)
Lesbian/Bisexual	2 (7.7%)
Relationship status	
Single	14 (53.8%)
Dating/Partnered	9 (34.6%)
Married	3 (11.5%)
Highest educational attainment	
Less than high school	6 (23.1%)
High school/equivalent	10 (38.5%)
Some college	8 (30.8%)
College graduate/higher	1 (3.8%)
Unknown	1 (3.8%)
Living situation	
Residential treatment program/sober house	6 (23.1%)
Room, apartment, house that I own or rent	13 (50.0%)
Shelter	1 (3.8%)
Transitional stabilization services	1 (3.8%)
With family or friends	4 (15.4%)
Unknown	1 (3.8%)
MOUD at delivery	
Discontinued MOUD during pregnancy	1 (3.8%)
Active non-prescribed use without MOUD throughout pregnancy	1 (3.8%)
Receiving MOUD	24 (92.3%)
Buprenorphine (of 24 participants receiving MOUD at delivery)	16 (66.7%)
Methadone (of 24 participants receiving MOUD at delivery)	8 (33.3%)
CPS report filed at delivery	25 (96.2%)
CPS report screened in for investigation (of 25 participants with report filed at delivery)	24 (96%)
Child removal at some point between most recent delivery and interview	12 (46.2%)
Reunification with child prior to interview (of 12 participants who experienced child removal)	6 (50%)
Child removal in previous pregnancy	10 (38.5%)

Our analysis revealed three main themes: (1) mothers who deliver infants exposed to MOUD perceive mandated reporting for risk of abuse and neglect as discriminatory, unjust, and stigmatizing; (2) mandated reporting caused anxiety and stress and negatively impacted family health and wellbeing; and (3) medical decisions by pregnant and postpartum individuals with OUD were influenced by the statewide mandated reporting policy.

*Theme 1: Mothers who deliver infants exposed to MOUD perceive mandated reporting for risk of abuse and neglect as discriminatory, unjust, and stigmatizing.*

Mothers experienced the language (e.g., “abuse” and neglect”) used in CPS reports for MOUD as stigmatizing and unjust:

“I was lookin’ at my paper. They put ‘Neglect’ on there. Why would they label it like that? She didn’t have anything in her system when she was born, just the [buprenorphine].” (Participant 3, 34-year-old white mother)

Another participant reflected that the CPS report and its effects painted her with the identity of a “child abuser.” She felt it was unfair and discriminatory that her name was placed on the child abuse registry list (Table 2, quote 2). Her name was ultimately removed after she sent a letter requesting a correction. Participants perceived CPS reports as punitive and inappropriate when the only indication was prenatal MOUD utilization (Table 2, quote 3).

**Table 2:** Participant quotations

Theme 1	Quote 2	“They put my name on a registry of child abusers all for taking a medication that I wasn't allowed to get off of.” (Participant 4, 31-year-old white mother)
	Quote 3	“I definitely think that if … you have either done something in your history of parenthood to show that you are not fit to be a parent, or to show that you are not safe, or that you put your child in harm’s way … that warrants an investigation, or a case … In our situation, again, I’m not trying to sound boastful, but I literally did every single thing right from the second that I found out I was pregnant.” (Participant 12, 26-year-old white mother)
	Quote 4	“Yeah, both of the hospitals and [CPS] were aware that my husband was on the same treatment that I was on. Nobody filed … against him, right. Why? We’re both parenting here, right. Why is it? Why are they not filing … against men that are on [MOUD] and have newborns, right? It’s only against women, and I feel like that’s also gender discrimination too, right. We’re doing the same thing … it’s abuse and neglect for me to take something to treat my addiction. Yet it’s not for him.” (Participant 13, 40-year-old white mother)
Theme 2	Quote 6	“I was so scared. I had everything prepared for the baby… He had everything he needed. I wasn’t scared on that aspect, but I knew that [CPS] was gonna be involved. Having [CPS] involved is a scary situation. One of your children gets taken away from [CPS], you think the littlest thing that you do, they can go and take away your son.” (Participant 5, 27-year-old Latina mixed-race mother)
	Quote 8	“[My health care providers] agreed to let me stay on the [buprenorphine] during pregnancy. My entire pregnancy I was told that [CPS] would come do a brief investigation and then they would leave. That was not what happened to me. They opened the case on me … there was no reason for them to have gotten involved… How I’ve been treated because of being on [buprenorphine], having a child is absolutely horrifying.” (Participant 4, 31-year-old white mother)
	Quote 9	“I wanted to keep that stage or part of my family private … I was completely exposed to my whole entire family ‘cause [CPS] wanted a safety plan. I don’t have many friends over here in Boston … In that case, I was forced to include my family for my family to know what was going on.” (Participant 14, 40-year-old Latina mother)
	Quote 10	“Just the fact that some strange guy is showing up saying that he wants to meet with my son. I just thought it was really inappropriate … I don’t know what they expected from my four-year-old. Did he think that [my son] was gonna say, “Mommy and Daddy smoke crack” or whatever? I don’t know what his expectation was, but he interviewed my son.” (Participant 13, 40-year-old white mother)
Theme 3	Quote 15	“[The social worker] was like, ‘Well, before you leave, remember, you’re gonna have [CPS] in your life and they’re gonna [file a report]. You might wanna tell me what your plan is before you leave here’ cause I will have to document that you had no plan and [were] pregnant.’ … so… so I just stayed.” (Participant 21, 31-year-old Black mixed-race mother)
	Quote 16	“They would make comments like that like, ‘Wow, that’s really high. Are you ever thinking about going down? What’s your plans for after?’ … they’re always like, ‘Okay, what dose are you on?’ Then I feel like they’re almost expecting me to be going down. That’s the impression that I got.” (Participant 25, 35-year-old white mother)
	Quote 18	“That trickle-down effect goes to [CPS]. That’s how much it trickles down, that [CPS] is now gonna be in more than what it was when I was able to stay on the clinic. Now I have to explain myself to [CPS], all because [the methadone clinic] didn’t wanna follow the rules that the governor put in place, and I just asked them to do that. I just asked them to not put anybody near me when I’m dosing. How hard is that?” (Participant 15, 31-year-old Black mother)

Interviewees noted gender-based discrimination in addition to SUD discrimination inherent in the policy. One interviewee shared frustration that the father of her baby also received MOUD, but only she was subject to a CPS report (Table 2, quote 4). Finally, participants described that CPS report filings increased stigma in the healthcare setting and contributed to being seen as an unfit mother. Participant 1 described the judgement she felt from hospital staff following her delivery:

“I feel like she was very insensitive to what was going on. I just feel like just because [CPS] has to get called, doesn’t mean you are the most horrible person in the world … She was … lookin’ at me almost like, ‘Oh, she just had a baby. This lady must be f****d up because she has [CPS]—we need to get [CPS] in her life.’ It’s like, ‘No lady. You don’t know what I’ve been goin’ through the last nine months.’” (Participant 1, 28-year-old Latina mother)

*Theme 2: Mandated reporting caused anxiety and stress and negatively impacted family health and wellbeing.*

Several participants discussed the intense anxiety they felt during pregnancy knowing that they would be reported to CPS at delivery. One mother with a previous child removal reported feeling prepared for her baby in all aspects except for CPS involvement (Table 2, quote 6). A CPS report filing can trigger anxiety and stress even when a case is not ‘screened in’ for further investigation. One participant who had a report ‘screened out’ explained that the experience made her retrospectively doubt her decision to continue MOUD during pregnancy:

“[CPS] coming into our life, even if it’s for a second, or even if it’s for a minute, it makes you think, “Wait a second.” You don’t know how many hours I’ve spent thinking, “What could I have done different? What could I have done to make it so that this letter wasn’t written?” … What could I have done to make it so that the hospital didn’t have to submit this one thing … saying that I put my child in danger? Should I have stopped taking [my medication] and then hurt her, her as a fetus?” (Participant 12, 26-year-old white mother)

Several participants described frustration at the disconnect between prenatal counseling to remain on MOUD during pregnancy and DCF actions. One mother described angst when a case was opened after being advised during pregnancy that CPS would likely screen the report out (Table 2, quote 8).

Interviewees also experienced anxiety from loss of privacy. Reporting can open families to invasive investigations; one mother described that due to CPS involvement, she was forced to disclose her SUD diagnosis and treatment to her whole family, despite her desire to keep this medical information private (Table 2, quote 9). Another mother reported that a caseworker showed up unannounced to her older child’s daycare to question him individually as part of the investigation for her newborn’s case (Table 2, quote 10). She found this inappropriate as it could have resulted in disclosure of medical information to school staff.

An open CPS case can affect many aspects of family health and wellbeing. The same participant reported anxiety while her daughter’s case remained open which exacerbated her trauma from a previous custody loss. She shared a conversation she had with her CPS caseworker:

“‘I don’t think you understand what having this open feels like to me. I’m concerned that I’m gonna go pick up my son at daycare, and you’re gonna have taken custody. I’m concerned that I’m gonna go back to the [hospital], and I’m not gonna be allowed to see my daughter… [the CPS worker] is like, ‘No, no. That’s not what any of us are thinking, or we’re not doing that.’ It’s like, ‘Great, but I don’t f*****g trust you.’ That’s what I believe is going to happen so, it’s causing a lot of anxiety as I’m trying to manage a lot of other things … He still dragged his feet for five weeks.” (Participant 13, 40-year-old white mother)

Those who lost custody at or following delivery (12 of the 26 interviewees) reported trauma from the experience, even if they were reunited with their children. One mother, who used non-prescribed substances throughout her pregnancy and was later reunited with her children in a family residential treatment program, recalled the acute pain she experienced when she was informed that her children were to be removed from her custody:

“It was just the worst pain of my life. It was like somebody stabbing me in my heart. That was the worst pain in my life. I surrendered that day. I was just like, ‘I am so done.’ …. I was so hurt.” (Participant 8, 24-year-old Black Latina mother)

Another mother described the impact of CPS taking custody while her daughter was still admitted to the hospital on her ability to care for her newborn. Though a judge granted her custody a week later, she was initially not allowed to visit her infant in the hospital:

“She’s only three days old. And, you know, I’m not comin’ to spend time with her. I’m not breastfeeding her. I’m not doing what I need to do as a mom. That’s craziness.” (Participant 21, 31-year-old Black mixed-race mother)

*Theme 3: Medical decisions by pregnant and postpartum individuals with OUD were influenced by the statewide mandated reporting policy.*

Mandated reporting and CPS recommendations impacted treatment decisions for some mothers. One participant who was treated with buprenorphine for two years prior to her pregnancy discontinued her medication during pregnancy to avoid CPS involvement, against her obstetrician’s recommendation:

“They’ll say, ‘oh, but you’ve been in treatment the whole time. You’ve had [expected toxicology tests] the whole time. It would be case closed.’ I was like, no. I don’t want a paper trail like that. I’d rather just stop using [MOUD] while I’m pregnant … [CPS] was just a huge fear ‘cause I was like, I've never been involved with them. I don’t want that on my record.” (Participant 9, 32-year-old Black mixed-race mother)

Another mother felt that the hospital social worker used information about CPS involvement post-delivery to coerce her into staying at the hospital for methadone titration during her pregnancy despite discomfort with her care. She described that when she began to leave, the social worker threatened to communicate to CPS that the participant left without a treatment plan after delivery (Table 2, quote 15).

Several participants noted that their CPS caseworkers gave medical guidance, often reflecting stigma against MOUD. One mother felt that her CPS caseworker consistently suggested that she lower her methadone dose (Table 2, quote 16). Another mother, who was prescribed clonazepam and methadone, said that she was instructed by her CPS caseworker to stop one of the two medications:

“Like after being on them for so long, it’s kind of hard to just come off… I think at that point, they were just grasping for straws. They wanted me to lose my daughter … I chose to come off the [clonazepam], and it’s not an easy detox, and I ended up relapsing … I had been sober for that whole time, and then, of course, I ended up relapsing and I lost her.” (Participant 24, 35-year-old white mother)

Furthermore, some interviewees explained how challenges engaging in SUD treatment impacted CPS cases. One participant who was discharged from her methadone clinic after an altercation about the clinic’s compliance with COVID-19 safety measures expressed frustration that her discharge would impact her CPS case (Table 2, quote 18). Finally, some participants believed that getting care at a specialized clinic for pregnant and postpartum individuals with SUD put them at increased risk for CPS involvement. One mother said that she believed CPS had an “umbrella” over the clinic where she received care and expressed regret about her choices:

“People are saying, ‘If I would have never started this [medication] or I came to this clinic, [CPS] would have never came into my life or taken my child away from me.’ It's looking really bad when [the clinic is] so great.” (Participant 14, 40-year-old white mother)

## Discussion

Our analysis details the detrimental impacts of mandated reporting for prenatal MOUD utilization. Participants experienced anxiety and stress which harms the health of the maternal-infant dyad, and increases risk of treatment nonadherence, return to non-prescribed substance use, and overdose (Davis et al., 2011; Thumath et al., 2021; Wadhwa et al., 2011). Participants felt that this policy reinforces the stigma that pregnant people with SUD are unfit mothers and exemplifies how reproduction is differentially encouraged across groups (Ross & Solinger, 2017). Our findings suggest that mandated reporting for prenatal MOUD exposure is not a benign intervention to prevent potential future harms to children, but tangibly harms parents and children.

In Massachusetts law, “physical dependence upon an addictive drug at birth” is classified as physical injury to a child (“Mass. Gen. Laws Ann. ch.119 § 51A,” 2020). This policy conflates MOUD receipt with child abuse because in-utero MOUD exposure can result in NOWS—a transient and treatable condition. CPS reporting algorithms for behaviors during pregnancy rely on harmful, patriarchal notions of “fetal rights” versus the rights of pregnant people, often deployed to limit reproductive freedoms (Goodwin, 2014). Notably, while several classes of maternal medications including benzodiazepines, antidepressants, and betablockers can transiently affect neonates, only MOUD is targeted in CPS reporting guidelines (Massachusetts Department of Children & Families, 2016). Participant experiences with CPS reporting and characterization as unfit mothers reflect societal attitudes toward and regulation of motherhood among people who use or have used drugs (Kenny & Barrington, 2018). Participants further noted that Massachusetts policy, which does not compel CPS reporting against fathers receiving MOUD, constitutes gender discrimination. It is crucial that policies support autonomous reproductive decisions without discrimination based on gender or SUD history.

Mandated reporting policies for newborns exposed to MOUD are ostensibly designed to identify infants at risk for abuse and neglect to prevent harm. However, we identified that reporting itself caused tangible harm due to the trauma caused by an investigation of parental fitness in the immediate postpartum period (Merritt, 2020; Zeman, 2004). This policy approach can harm the whole family in order to avoid theoretical risk of future harm to the infant (Broadhurst & Mason, 2019; Howard et al., 2011; Kenny et al., 2015) and prioritizes child “protection” at the expense of maternal and family wellbeing. Further, in instances where ongoing CPS involvement followed mandated reporting, pregnant and parenting people with SUD felt stripped of their right to make decisions about their own bodies and reproduction. Participants reported making decisions primarily to appease or avoid CPS, sometimes to the detriment of their health, recovery, and/or children. Fear of CPS involvement is well documented as a barrier to care and services for pregnant people with SUD (Stone, 2015; S. C. Roberts & Pies, 2011); our analysis found that the Massachusetts reporting policy also affected the treatment decisions of the women in our study who sought prenatal care. The finding that participants’ CPS caseworkers commented on mothers’ medical and behavioral care and that this influenced participants’ medication decisions is concerning. Medical decisions are personal and complex and should be made autonomously with a knowledgeable healthcare provider—not out of fear of CPS involvement.

This study had several limitations. First, we conducted interviews both prior to and during the COVID-19 pandemic and the experience of COVID and the interview modality (in person or by phone) may have influenced responses. Second, because this is a secondary analysis from a study about experiences with MOUD, we were unable to explore experiences of pregnant people using non-prescribed substances only. Additionally, most of the participants in this study were seen at a multidisciplinary clinic specialized in caring for families affected by SUD which may affect generalizability. Finally, while several participants of color reported experiences that may have been influenced by racism in the child welfare and medical systems—which disproportionately harm families of color (Dettlaff & Boyd, 2020; Roberts, 2022)—the interview guide did not investigate the impact of racism on participants’ experiences. Future research should specifically address the impact of racism on experiences of dyads affected by OUD and CPS involvement.

To ensure the safety and improve health outcomes for maternal-infant dyads, MOUD treatment decisions made by pregnant people must be uncoupled from CPS reporting. Several states have established policies that use de-identified notifications for newborns exposed to prescribed opioids for federal epidemiologic tracking and require CPS reports only for non-prescribed prenatal substance use to minimize harms of extraneous reporting (Connecticut Department of Children & Families, 2018; Rhode Island Department of Children, 2018). In other states, CPS notifications are made regardless of legality or prescription of substances primarily to provide care coordination, but a report of abuse/neglect is only made for safety risks other than in-utero substance exposure (New Hampshire Center of Excellence on Addiction, 2019; “New Mexico Ann. Stat. § 32A.3A.13,” 2019). These pathways illustrate alternative policy options for meeting current federal requirements without requiring CPS reports for prenatal MOUD utilization.

Most importantly, clinicians caring for families impacted by substance use should focus on service delivery, not surveillance for CPS. Centering interventions on concrete assistance for parental and infant health and building parenting skills may best support the health of families affected by SUD. Policies that promote community care (Rise Participatory Action Research Team, 2021) and support pregnant and postpartum people with services such as parent–child home visiting, childcare, and maternal-infant mental health programs are supportive alternatives to mandated reporting.

## Supplementary Information

Below is the link to the electronic supplementary material.

Supplementary file1 (DOCX 30 KB)

## Data Availability

N/A.

## Code Availability

N/A.
