# Supplementary material for: Prescribed and Penalized: The Detrimental Impact of Mandated Reporting for Prenatal Utilization of Medication for Opioid Use Disorder
Source: Matern Child Health J. 2023 May 31;27(Suppl 1):104–12. doi: 10.1007/s10995-023-03672-x (PMC10229393; doi:10.1007/s10995-023-03672-x)
Supplement: Supplementary file 1 — Supplementary file1 (DOCX 30 KB) [file 10995_2023_3672_MOESM1_ESM.docx]

MOUD Interview Guide

*Thank you for taking the time to participate in this interview. As I described a bit before, the goal of this interview is to really get to know you in order to better understand your experiences, your background, and all the different parts of you that make you who you are.*

*We are interested in really getting to know you as a person, so that we can best understand your experiences around receiving treatment for your opioid use disorder. We hope to have an honest conversation about how your experience with this treatment during and after pregnancy. The answers you provide, positive and negative, will help us improve care in our clinic.*

*With that, I’d like to start by asking you some initial questions to learn more about you.*

| **Question** | **Follow-Up/Probes** |
| --- | --- |
| Can you start off by telling me a little bit about yourself? | What should I know about you to see who you really are.  Can you tell me about how some of your life experiences or other formative things about you? |
| Does religion play an important role in your life? | What role does it play? |
| In the culture you grew up in, how was drug use viewed? | You can define culture in however it means to you, including the beliefs, attitudes, and values in the community you were raised |

*Thank you for sharing that with me. Now I would like to ask you a bit about some of your treatment experiences.*

| **Question** | **Follow-Up/Probes** |
| --- | --- |
| How did you find yourself needing medical care and medication for substance use? | What types of treatments have you tried in order to stop using opioids?  What did and did not work for you in your treatment for your opioid use disorder? |
| What medication (if any) are you taking now for your opioid use disorder? | How long have you been taking either buprenorphine or methadone medication treatment?  Did you begin this treatment during pregnancy at the suggestion or your doctor? Or did you continue the treatment you were on before? |
| How often do you take your medication? | Do you ever have leftover doses of your medication?  If you have ever had leftover doses, what did you do with them? Have you ever shared, exchanged, or sold your medication to someone else? |
| Can you tell me about your experience(s) taking medication for your opioid use disorder? | What was it like to start treatment? What were the effects it had in your life? |
| What was your experience taking this medication during your pregnancy? | Did your dose or frequency of doses change while you were pregnant?  How did you feel taking your medication during pregnancy?  Did you want to increase your dose or cut back for any reasons? |
| How did you feel about being in “control” while using this medication?  (You can define control as a reduction in withdrawal symptoms and cravings, improvement in your ability to function.) | Did this medication help you to feel more in control while using it?  Did you feel it took away your sense of control, added to your sense of control, or had no impact on your sense of control?  What was the effect of this medication on your withdrawal symptoms or craving? |
| Do you take any medications for your mood or anxiety? | How difficult is it to take those medications routinely? |

*Thank you again for sharing this with me. Now I want to talk with you more about what happened after your delivery and with your newborn baby. If your delivery was impacted by COVID, I would like to hear about your experiences with that as well.*

| **Question** | **Follow-Up/Probes** |
| --- | --- |
| How did you feel about your medication in the first few days to weeks after you delivered your baby? | We know that many women have some difficulty getting the right dose of their medication post-delivery. What was your experience with this?  Did you notice any physical changes related to your medication postpartum?  Did you notice any emotional changes related to your medication postpartum?  Have you made any changes to your medication dosing after delivery? |
| Have there been any challenges in continuing your medication in the postpartum period? | Can you describe those challenges?  Parenting? Working? residential treatment program requirements? Moving? |
| What feelings did you have around your baby’s experience in the hospital? | Did you have any negative feelings surrounding your baby’s withdrawal or hospital stay?  What triggered those feelings? What helped/has helped to minimize them? What can we do as a healthcare team to help mothers either avoid or manage any negative feelings? |
| Did you observe any withdrawal signs in your baby after delivery? | If so, how did you react to these signs?  At the time you noticed the baby going through withdrawal, what did it bring up for you in terms of your own experiences of withdrawal?  Did your baby require treatment with medicine for withdrawal symptoms? If so, did you feel involved in that decision? Please tell me more about that. |
| Have you experienced any positive feelings around how you have managed your substance use during your pregnancy or care for your baby after birth? | If yes, what has led to those feelings? Can you recall any specific interactions or things somebody said?  How can we as a healthcare team help support and highlight your accomplishments? |

*Now I’d like to ask you about your personal attitudes/views of your medication treatment.*

| **Questions** | **Follow-Ups/Probes** |
| --- | --- |
| What were/are some things that you liked about taking buprenorphine or methadone? | What made it easy to continue taking this medication? |
| What were/are some things that you dislike about taking buprenorphine or methadone? | What made it difficult for you to continue taking this medication? |
| What in your upbringing might have influenced your decisions surrounding medication treatment? | Did your religious beliefs or cultural background play a role in your ideas and decisions around medication treatments? |

*Now I’d like to understand a bit more about those closest to you view medication treatment.*

| **Questions** | **Follow-Ups/Probes** |
| --- | --- |
| What does your family know about your treatment (with buprenorphine or methadone)? | What are their thoughts about your medication use? What was their reaction when they found out you were receiving medication treatment? |
| Have the views and thoughts of your family impacted your decision to take or not take your medication treatment at any point (past or present)? | If so, how? |
| What do your close friends know about your treatment (with buprenorphine or methadone)? | What are their thoughts about your medication use? What was their reaction when they found out you were receiving medication treatment? |
| Have the views and thoughts of your close friends impacted your decision to take or not take your medication treatment at any point (past or present)? | If so, how? |
| Do you currently have a partner or are in a significant relationship? | If not currently, were you in a relationship/did you have a partner at the start of your last pregnancy? |
| What were your partner’s thoughts on your medication (buprenorphine, methadone) use at that time? | What was their reaction when they found out you were receiving medication treatment? |
| Have the views and thoughts of your current or previous partner impacted your decision to take or not take your medication treatment at any point (past or present)? | If so, how? |
| Does/did this partner have a history of substance use disorder themselves? | Have they ever been on medication treatment? |
| Can you tell me about your current living situation? | Do you live in some kind of housing which you do not personally own or rent? Residential treatment? A shelter? Public housing? Staying with family/friends? |
| How does the place you reside affect your ability to take your medications? | Do you have any difficulty taking your medications as scheduled?  How those you live with or who manage your housing understand your medication treatment? |

*We’re a little more than halfway through the questions I’d like to ask you. I know we are asking you to discuss sensitive topics or recall upsetting memories, so please let me know if you’d like to take a break.*

*If you’re ready to continue, I’ll now ask you questions about your experiences with treatment, broadly speaking.*

| **Questions** | **Follow-Ups/Probes** |
| --- | --- |
| Are there any social supports that help you maintain your recovery? | If yes, can you tell me a little bit about them. |
| Tell me about how your healthcare provider speaks with you about your substance use treatment? | How important is your providers’ approval and support to you? |
| If you have cravings or a desire to use, who do you turn to? | What does that person do to support you? |
| What other types of recovery supports do you use other than medication treatment? | Do you attend an intensive outpatient program (IOP)? Do you attend AA or NA or Smart Recovery meetings?  Do you engage in individual therapy?  Do you live in a residential treatment program? |
| To what extent do you take your medications as directed each day (this could mean your buprenorphine, methadone, or any other medications you are prescribed)? | What helps you stay on top of taking your medications each day? |
| What are some reasons you have stopped (or had difficulty with) your medication treatment in the past? | Did you ever encounter issues at the pharmacy or methadone clinic?  Did you find it difficult to continue attending regular appointments/meeting the requirements of a program?  Were there other reasons you discontinued your medication treatment? |
| What have your experiences been like with health care providers around your substance use disorder? | Specifically, what positive experiences have you had?  How about what negative experiences you had had? |
| Do you currently have an open case with DCF? If not, do you have previous involvement with DCF? |  |
| If so, can you tell me a bit about your DCF caseworker? | Do/did they know about your medication treatment?  What do/did they think about/how did they view your medication treatment? |
| Do you currently receive services from Early Intervention? | Does your EI provider know about your medication treatment?  What do they think about/how do they view your medication treatment? |
| Are you currently on parole or in a drug court program? | Does your parole office know about your medication treatment?  What do they think about/how do they view your medication treatment? |
| Do you receive any other social services? | Do you receive home visiting services, or an intensive outpatient program?  If so, do the service providers you interact with from there know about your medication treatment?  What do they think about/how do they view your medication treatment? |

*Now I’d like to ask you about what supports or parts of treatment you find do or don’t support your recovery, and to better understand how you know your treatment is succeeding/working, or what encourages you to stay engaged in treatment.*

| **Questions** | **Follow-Ups/Probes** |
| --- | --- |
| What are things you think are most important to you maintaining your recovery? |  |
| Are there things that support your recovery that a clinic (like HOPE, RESPECT, etc.) might be able to offer you? | What would those things look like? What kinds of support specifically would be helpful? |
| If you can think back to any treatments, clinics, programs you have been to what did you like and dislike about these? | What about the treatment program (requirements, structure, design) was helpful or unhelpful to you?  Did you ever feel you were treated unfairly by program staff or healthcare providers for any reason?  Can you identify the reason (race, gender, class)? |
| What are skills that you think are important to work on during treatment and recovery? |  |
| What additional support do you think would be helpful after delivery that is different from when you were pregnant? |  |
| Tell me what success means for you during drug treatment? | What would some of the smaller successes be for you along the way? |
| How do you know when your medication treatment is working? |  |
| How do you know when other aspects of your treatment are working? |  |

*Thank you again for sharing, we are learning a lot from you answers and are nearing the end of the interview. One of our last set of questions asks about the experiences you may have faced while parenting while on medication.*

| **Questions** | **Follow-Ups/Probes** |
| --- | --- |
| Can you remind me, is your baby living with you now? |  |
| Do you have any fears or concerns about parenting your child while continuing to care for yourself and manage substance dependence? | Can tell me more about that? |
| What parenting/child-rearing supports do you have? |  |
| How can we as a healthcare team for you and your baby help support your parenting strengths? |  |

*Incentives for maintaining treatment adherence:* Some research in pregnant women with opioid use disorder shows that different material supports, like money or supplies or food, can be used to motivate a woman in their recovery.

| Questions | Follow-Ups/Probes |
| --- | --- |
| What kind of things would motivate you to remain on your medication treatment after delivery? | [If custody of the baby] Would diapers, baby clothes, and other supplies be valuable?  [If does not have custody] Would working to arrange increased supervised visits with DCF be valuable? Would other incentives (materials, etc.) be valuable/helpful? |
| What kind of supports would help you [continue to maintain on/re-engage in] medication treatment after delivery? |  |

*Now I’d like to ask you some questions about your substance use treatment in the context of the ongoing COVID pandemic and the many impacts it has had on day-to-day life.*

| **Question** | **Follow-Ups/Probes** |
| --- | --- |
| How has COVID-19 impacted your addiction treatment and recovery? | If you live in residential treatment, have there been changes there that have impacted you? In what ways?  If you are on methadone treatment, have changes at the clinic impacted you? In what ways? (i.e: less frequent attendance, less frequent appointments with your counselor/clinician, etc.)  If you are on buprenorphine, how has coming to clinic less frequently impacted you? |
| What has it been like to see your providers mostly virtually/by phone? | Has less frequent toxicology testing impacted your recovery? |

*Preparing for and caring for a new baby can already be a stressful experience on its own. Many people are experiencing additional stress and anxiety due to fears about becoming ill or loneliness in social isolation. These next questions will ask a bit about how your experience of parenting has been impacted by COVID.*

| **Question** | **Follow-Ups/Probes** |
| --- | --- |
| Could you start by telling me what social distancing has looked like to you? | Have you made any changes to your daily routing because of COVID-related precautions?  Has this impacted your recovery? Can you describe in what ways? |
| Can you tell me about any challenges social distancing has presented in providing care your baby? | Do you have another caregiver or support person in your household to help care for your baby? What other resources for support are you able to access?  Have you or anyone in your family/home become ill with COVID-19? How has that impacted your ability to care for yourself (and/or your baby)? |
| Have you experienced income loss or other financial instability because of the Coronavirus pandemic and social distancing measures? | If so, please tell me more about how this has impacted your family? In what ways has any new financial insecurity impacted your recovery?  Are you having difficulty accessing material supplies like diapers, wipes, formula, etc.? |

*Now I’d like to ask you some questions about your experience with DCF in the context of COVID and COVID precautions.*

| **Question** | **Follow-Ups/Probes** |
| --- | --- |
| What is the status of your DCF case (investigation, open case, plan for reunification, custody arrangement, etc.)? | Has the status or plans for your case changed due to COVID?  How have caseworkers or investigators communicated to you during this time?  If you are not currently parenting, how has COVID changed your visitation schedule, plans for reunification, or other decisions about your case? |

*This marks the conclusion of the interview. We thank you so much for your time. I’ll now turn off the audio recorder.*
